# Adipocytokines in Untreated Newly Diagnosed Rheumatoid Arthritis: Association with Circulating Chemokines and Markers of Inflammation

**DOI:** 10.3390/biom11020325

**Published:** 2021-02-21

**Authors:** Georgios K. Vasileiadis, Anna-Carin Lundell, Yuan Zhang, Kerstin Andersson, Inger Gjertsson, Anna Rudin, Cristina Maglio

**Affiliations:** 1 Department of Rheumatology and Inflammation Research, Institute of Medicine, The Sahlgrenska Academy at University of Gothenburg, 413 46 Gothenburg, Sweden; georgios.vasileiadis@gu.se (G.K.V.); anna-carin.lundell@rheuma.gu.se (A.-C.L.); yuan.zhang@gu.se (Y.Z.); kerstin.andersson@microbio.gu.se (K.A.); inger.gjertsson@rheuma.gu.se (I.G.); Anna.Rudin@microbio.gu.se (A.R.); 2 Wallenberg Centre for Molecular and Translational Medicine at the University of Gothenburg, 405 30 Gothenburg, Sweden

**Keywords:** adipocytokines, adiponectin, chemokines, inflammation, rheumatoid arthritis

## Abstract

Adiponectin, leptin, and resistin are adipocytokines whose levels are elevated in blood and synovial fluid from patients with rheumatoid arthritis (RA). However, their role in RA pathogenesis is unclear. Here, we examined whether adipocytokines are associated with circulating chemokines, markers of inflammation and RA disease activity in patients with untreated newly diagnosed RA. Plasma levels of 15 chemokines, adiponectin, leptin, and resistin were measured using flow cytometry bead-based immunoassay or enzyme-linked immunosorbent assay (ELISA) in a cohort of 70 patients with untreated newly diagnosed RA. Markers of inflammation and disease activity were also assessed in all patients. Positive association was found between total adiponectin and CXCL10 (β = 0.344, *p* = 0.021), CCL2 (β = 0.342, *p* = 0.012), and CXCL9 (β = 0.308, *p* = 0.044), whereas high-molecular weight (HMW) adiponectin associated only with CXCL9 (β = 0.308, *p* = 0.033). Furthermore, both total and HMW adiponectin were associated with C-reactive protein (β = 0.485, *p* = 0.001; β = 0.463, *p* = 0.001) and erythrocyte sedimentation rate (β = 0.442, *p* = 0.001; β = 0.507, *p* < 0.001). Leptin and resistin were not associated with plasma chemokines, markers of inflammation, or disease activity scores. Our study shows an association between circulating adiponectin and pro-inflammatory chemokines involved in RA pathogenesis as well as markers of inflammation in a well-characterized cohort of patients with untreated newly diagnosed RA.

## 1. Introduction

Adiponectin, leptin, and resistin are cytokines produced by the adipose tissue, so-called adipocytokines or adipokines. Adipocytokines play regulatory roles in both metabolism and inflammation and they have been associated with rheumatoid arthritis (RA), a chronic, inflammatory, autoimmune disease mainly affecting the joints [1,2,3,4,5]. In fact, levels of adiponectin, leptin, and resistin have been reported as elevated in both blood and synovial fluid of patients with RA compared to controls [6,7,8,9,10]. It is still unknown if this is a mechanism of the adipocytokines to counterbalance the surrounding inflammation or if they are able to promote inflammatory changes leading to RA.

RA development is characterized by a pre-clinical state, in which the patients are asymptomatic but the autoimmune and inflammatory processes leading to the disease have already been initiated. Autoantibodies, cytokines, chemokines, and inflammation markers have been reported to increase in blood several years before the first signs of RA [11,12,13]. Chemokines are a class of small chemotactic cytokines implicated in the early phases of RA pathogenesis by promoting local and systemic inflammation and by recruiting leucocytes in the synovium [14]. Several chemokines, such as CCL2, CCL3, CCL4, and CXCL10, are elevated in both plasma and synovial fluid of patients with RA compared to controls. CXCL10 has also been suggested as a biomarker of disease activity in patients with RA [15,16].

Increased circulating adiponectin and C-reactive protein (CRP) levels precede the development of RA in patients with obesity [17]. Recombinant adiponectin is able to stimulate the production of pro-inflammatory chemokines and cytokines from peripheral blood mononuclear cells (PBMCs) and fibroblast-like synoviocytes (FLS) obtained from individuals without any known inflammatory disease [18]. Taken together, these results raise the hypothesis that adiponectin is implicated in the early phases of RA pathogenesis. However, a recent meta-analysis found no association of circulating adiponectin levels with CRP levels or with disease activity based on the composite index Disease Activity Score in 28 joints (DAS28) in patients with RA [19]. Adiponectin circulates in blood in three forms whereof the high-molecular weight (HMW) form is thought of as the active one in the context of metabolism [20]. It was recently shown that both total and HMW circulating adiponectin associate with CRP levels in patients with early RA [21].

Other adipocytokines, such as leptin and resistin, have also been associated with markers of inflammation and RA disease activity, although with inconsistent results [8,22,23,24,25]. A meta-analysis found circulating leptin levels to be correlated with CRP levels and DAS28 in patients with RA [25]. Additionally, serum resistin levels have been reported as associated with CRP, erythrocyte sedimentation rate (ESR), and DAS28 in RA patients [8,22]. Conversely, several articles have demonstrated no associations between leptin or resistin and markers of RA activity [23,24]. A reason for the discrepancy in the results regarding the association between adipocytokines and markers of disease activity in RA could be that most previous studies were performed in patients with established RA, without accounting for treatment or disease duration. Little is known about the association of adipocytokines with markers of inflammation and RA disease activity in untreated newly diagnosed RA [21,26,27]. Contradictory results have also been reported on the association between adipocytokines and markers of inflammation and disease progression in other rheumatic joint diseases, such as spondyloarthritis or psoriatic arthritis [28,29,30].

The aim of this study was to investigate whether circulating total and HMW adiponectin, leptin, and resistin levels were associated with plasma chemokines, markers of inflammation, and disease activity markers in a cohort of 70 patients with newly diagnosed untreated RA. Our results show that levels of total and HMW adiponectin, but not leptin or resistin, associated with pro-inflammatory chemokines as well as ESR and CRP in this patient group.

## 2. Materials and Methods

### 2.1. Patient Cohort

The patient group consisted of 70 treatment-naïve patients with newly diagnosed RA. Diagnosis of RA was made in accordance with the American College of Rheumatology/European League Against Rheumatism 2010 criteria. Inclusion criteria were > 18 years of age, rheumatoid factor (RF)-positivity and/or anti-citrullinated protein antibodies (ACPA)-positivity and/or CRP ≥ 10 mg/L, disease activity based on DAS28-CRP > 3.2 and > 2 swollen joint count (SJC) and tender join count (TJC) based on a 66/68 joint count. Other inclusion criteria were symptom duration of less than 24 months, absence of previous treatment with disease modifying anti-rheumatic drugs (DMARDs) or corticosteroids, and a good general health. Collection of blood samples was performed before treatment with DMARDs or corticosteroids started. Patient recruitment was carried out at the Rheumatology Clinics of Sahlgrenska University Hospital in Gothenburg and Skåne University Hospital (Sweden). All participants gave their written consent and study approval was given by the regional ethics committees of Gothenburg and Lund, Sweden (ethical approval number: 691–12, amendment number: T270–13).

### 2.2. Clinical and Biochemical Assessment

For every patient, swollen (66) and tender joint (68) count was performed and CRP and ESR were measured. DAS28 based on 28 joints and Clinical Disease Activity Index (CDAI) were calculated for each patient. Smoking was defined as current daily smoking. Multiplexed anti-CCP test (BioPlex, BioRad, Hercules, CA, USA) was used for the demonstration of ACPA positivity and nephelometry (Beckman Coulter, Brea, CA, USA) for RF positivity. ACPA- or RF-positivity was defined according to cut-off levels in the local clinical laboratories.

### 2.3. Assessment of Adipocytokines

Plasma was isolated from whole blood with the use of Lymphoprep (Axis-Shield, Oslo, Norway). Total and HMW adiponectin, leptin, and resistin in plasma were measured using the following enzyme-linked immunosorbent assays (ELISA): Human Total Adiponectin/Acrp30 Quantikine ELISA, Human HMW Adiponectin/Acrp30 Quantikine ELISA, Human Leptin Quantikine ELISA, and Human Resistin Quantikine ELISA (R&D Systems, Bio-Techne, Minneapolis, MN, USA). The experiments were performed according to the manufacturer’s instructions.

### 2.4. Assessment of Chemokines

The evaluation of pro-inflammatory chemokines in plasma was performed with the use of bead-based immunoassay, LEGENDplex™ Human Proinflammatory Chemokine Panel (BioLegend, San Diego, CA, USA) or with DuoSet ELISA (R&D Systems), according to manufacturers’ instructions. The bead-based panel was used for the measurement of CCL2 (MCP-1), CCL5 (RANTES), CXCL10 (IP-10), CCL17 (TARC), CCL3 (MIP-1α), CCL4 (MIP-1β), CXCL9 (MIG), CCL20 (MIP-3α), CXCL5 (ENA-78), CXCL1 (GROα), CXCL11 (I-TAC), and CXCL8 (IL-8). A FACSVerse equipped with FACSuite software (BD Biosciences, San Jose, CA, USA) was used for sample acquisition and the FCAP Array software (Soft Flow, Pecs, Hungary) for data analysis. ELISA (DuoSet) was used for the measurement of CCL22 (MDC) and CXCL13 (BCL) since those two chemokines were not included in the above Multiplex set. CCL11 (Eotaxin) was also measured by ELISA since values given by the bead-based immunoassay were higher compared to previously published levels [15]. To assess possible false positive results in the analysis of chemokine and adipocytokine levels caused by heterophilic antibody interference, HeteroBlock (Omega Biologicals, Bozeman, MT, USA) was used in a subgroup of RF-positive patients. The detected levels of chemokines and adipocytokines with or without HeteroBlock treatment in RF-positive patients did not differ significantly (data not shown).

### 2.5. Statistical Analysis

Kolmogorov–Smirnov test was used to check for normality of the distribution for each variable. Since most of the variables did not follow a normal distribution, all variables were log-transformed in all analyses. Multivariate factor analysis orthogonal projection to latent structures (OPLS) was performed to identify associations between each adipocytokine (y variables) and clinical disease activity measures or concentrations of various chemokines in plasma (x variables) in linear multivariate models (SIMCA software; Sartorius Stedim Biotech, Umeå, Sweden). Resulting column bars represented the importance of each x-variable to the y-variable. The software used Jackknifing to calculate standard error displayed as an error bar on each column, which represented the 95% confidence interval. The data were scaled to unit variance in SIMCA by dividing each variable by 1/standard deviation, resulting in all variables being given equal weight regardless of their absolute value. The scale of the axes in OPLS is dimensionless and there is normalization of the loading vector to a length of one. For the quality appraisal of the OPLS models, parameters R2 (how well the variation of the variables is explained by the model) and Q2 (how well a variable can be predicted by the model) are presented for each OPLS model. Multiple linear regression analysis was performed using SPSS Statistics 25 (IBM, Armonk, NY, USA) to examine the relationship of each adipocytokine with clinical markers of disease activity and chemokines in patients with untreated newly diagnosed RA. Any chemokine or adipocytokine value below the detection limit, was included in the analysis with a value equal to half the detection limit. Graphs were made with GraphPad Prism version 8.2.0 (GraphPad Software, San Diego, CA, USA).

## 3. Results

### 3.1. Clinical Characteristics and Chemokine Measurement

Clinical characteristics of the cohort of 70 treatment-naive patients with newly diagnosed RA are described in Table 1. The cohort included mostly women (69%), with median age of 55 years, median body-mass index (BMI) of 25, and median patient-reported symptom duration of 5 months. The majority of patients were ACPA- and RF-positive. The patients included in the cohort had a median DAS28 score of 5, median CDAI of 28, median CRP of 9 mg/L, and median ESR of 24 mm/hour.

Total and HMW adiponectin, leptin, and resistin as well as 15 chemokines were measured in plasma of the RA cohort. Plasma concentrations of adipocytokines and chemokines are reported in Table 2. Median total adiponectin was 6.0 mg/L, median HMW adiponectin was 3.0 mg/L. Median leptin was 13.2 ng/mL and median resistin was 10.1 ng/mL. As a positive control, we tested whether the adipocytokines were associated with sex, age, and BMI as previously reported [31,32,33]. As expected, total adiponectin was significantly associated with BMI (β = −0.416, *p* value = 0.002), male sex (β = −0.371, *p* = 0.007), and age (β = 0.304, *p* = 0.030) while HMW adiponectin showed a significant association only with BMI (β = −0.401, *p* = 0.002). Leptin was significantly associated with BMI (β = 0.672, *p* < 0.001) and male sex (β = −0.655, *p* < 0.001), while resistin was only associated with male sex (β = −0.250, *p* = 0.049).

### 3.2. Association of Adipocytokines with Plasma Chemokines

**Figure 1:**
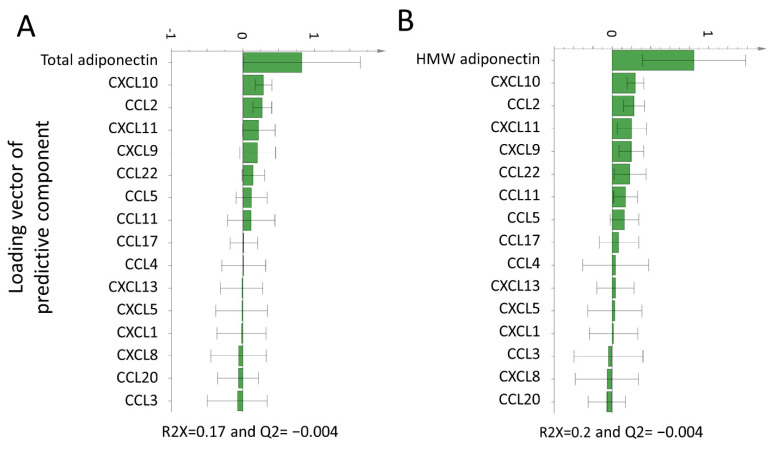
Orthogonal projection to latent structures (OPLS) loadings column plot of the association of total and high-molecular weight (HMW) adiponectin with plasma chemokines. OPLS models depicting the association of total (**A**) and HMW (**B**) adiponectin with plasma chemokines. X-variables represented by a bar pointing in the same direction as the y-variable (adiponectin) were associated with higher total or HMW adiponectin levels, whereas x-variables represented by a bar pointing in the opposite direction were related to lower total or HMW adiponectin. Only variables associated with plasma total/HMW adiponectin in the OPLS analysis were tested for significance with linear regression analyses. Abbreviations: HMW, high molecular weight; OPLS, orthogonal projection to latent structures.

We first investigated whether total and/or HMW adiponectin were associated with plasma chemokines in patients with newly diagnosed untreated RA. Both total and HMW adiponectin associated positively with CXCL10, CCL2, CXCL9, and CXCL11 based on the OPLS models shown in Figure 1. 

**Figure 2:**
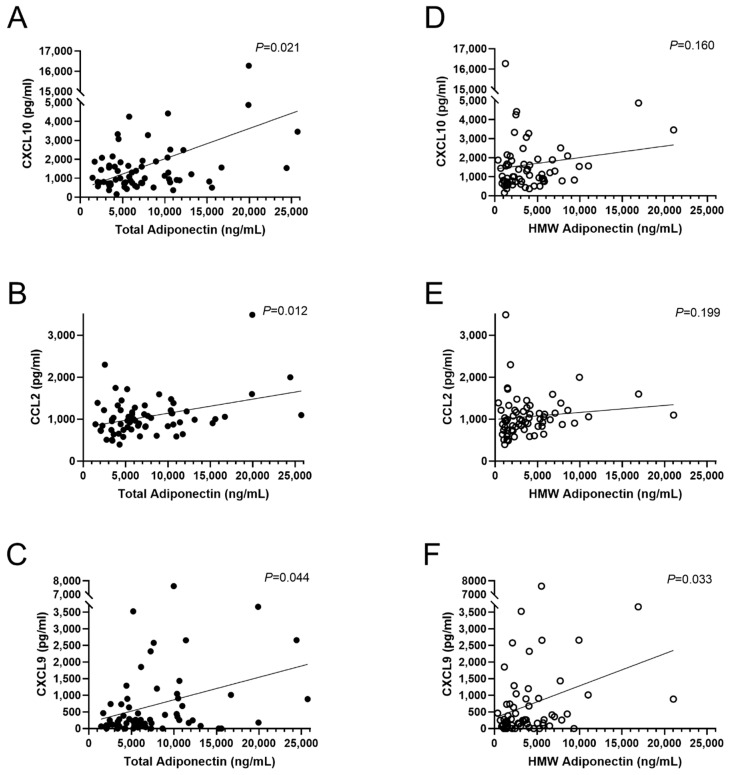
Multiple linear regression analysis of total and HMW adiponectin with plasma chemokines. (**A**–**C**) Linear regression analysis of total adiponectin with plasma chemokines. (**D**–**F**) Linear regression analysis of HMW adiponectin with plasma chemokines. Analyses were adjusted for sex, age, and BMI and were performed after log-transformation of all variants Abbreviations: BMI, body-mass index; HMW, high molecular weight.

Those associations were then tested by multiple linear regression analysis after adjustment for sex, age, and BMI. Levels of total adiponectin were associated with levels of CXCL10 (β = 0.344, *p* = 0.021), CCL2 (β = 0.342, *p* = 0.012), and CXCL9 (β = 0.308, *p* = 0.044), as shown in Figure 2. HMW adiponectin was only associated with CXCL9 but not CXCL10 and CCL2 (β = 0.315, *p* = 0.033, Figure 2). Total and HMW adiponectin were not associated with CXCL11 (β = 0.001 *p* = 0.999 and β = −0.037, *p* = 0.795, respectively).

In the OPLS models, leptin and resistin showed a positive association with several chemokines (Supplementary Figure S1). However, in multiple linear regression analysis after adjustment for sex, age, and BMI, no significant associations were found for either leptin or resistin with plasma chemokine levels (Supplemental Tables S1 and S2).

### 3.3. Association of Adipocytokines with Inflammation and Disease Activity Markers

**Figure 3:**
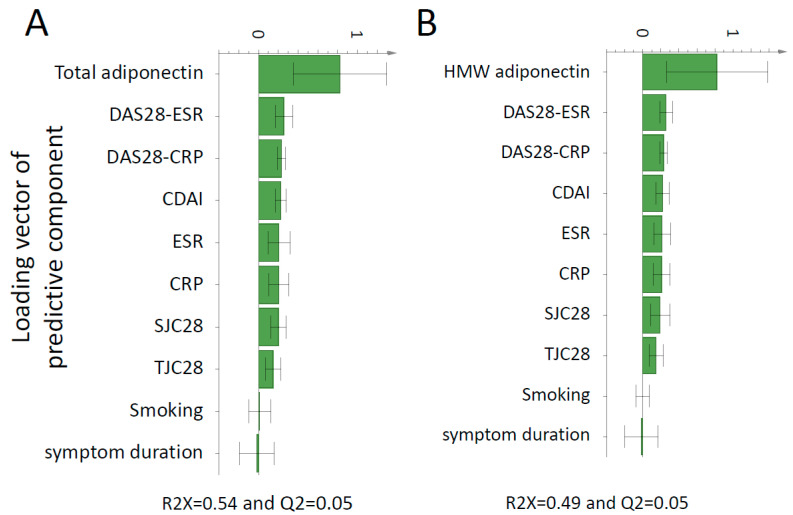
OPLS loadings column plot of the association of total and HMW adiponectin with clinical markers of disease activity. OPLS models depicting the association between total (**A**) and HMW (**B**) adiponectin with clinical markers of disease activity. X-variables represented by a bar pointing in the same direction as the y-variable (adiponectin) were associated with higher total or HMW adiponectin, whereas x-variables represented by a bar pointing in the opposite direction were related to lower total or HMW adiponectin. Only variables associated with plasma total/HMW adiponectin in the OPLS analysis were tested for significance with linear regression analyses. Abbreviations: CDAI, clinical disease activity index; CRP, C-reactive protein; DAS28, disease activity score in 28 joints; ESR, erythrocyte sedimentation rate; HMW, high molecular weight; OPLS, orthogonal projection to latent structures; SJC28, swollen joint counts of 28; TJC28, tender joint counts of 28.

**Figure 4:**
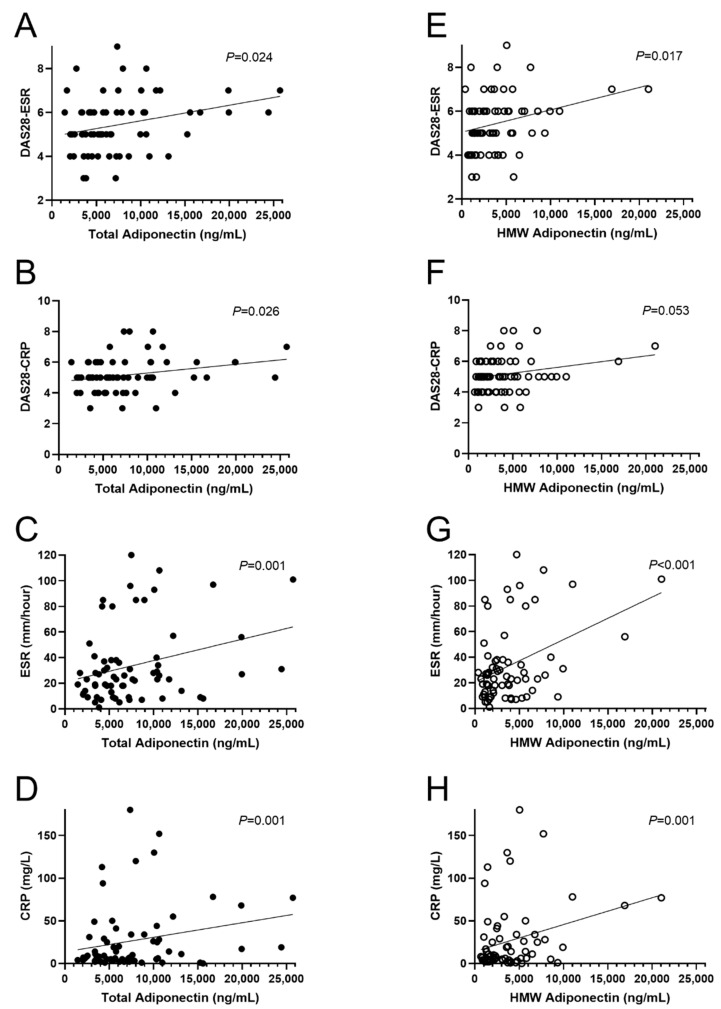
Multiple linear regression analysis of total and HMW adiponectin with clinical markers of disease activity. (**A**–**D**) Linear regression analysis of total adiponectin with clinical markers of disease activity. (**E**–**H**) Linear regression analysis of HMW adiponectin with clinical markers of disease activity. Analyses were adjusted for sex, age, and BMI and were performed after log-transformation of all variants. Abbreviations: BMI, body-mass index; CRP, C-reactive protein; DAS28, disease activity score in 28 joints; ESR, erythrocyte sedimentation rate; HMW, high molecular weight.

OPLS models demonstrated that both total and HMW adiponectin were positively associated with all inflammation and disease activity markers examined as shown in Figure 3A,B. As shown in Figure 4, in multiple linear regression analysis after adjustment for sex, age, and BMI, total adiponectin was positively associated with DAS28-ESR (β = 0.331, *p* = 0.024), DAS28-CRP (β = 0.335, *p* = 0.026), ESR (β = 0.442, *p* = 0.001), and CRP (β = 0.485, *p* = 0.001). Similarly, HMW adiponectin was positively associated with DAS28-ESR (β = 0.335, *p* = 0.017), ESR (β = 0.507, *p* < 0.001), and CRP (β = 0.463, *p* = 0.001) but not DAS28-CRP (β = 0.282, *p* = 0.053). Neither total nor HMW adiponectin were associated with CDAI (β = 0.165, *p* = 0.275 and β = 0.153, *p* = 0.293, respectively).

We also performed a multivariate OPLS analysis for leptin and resistin with inflammation and disease activity markers. The models obtained showed a weak positive association between leptin and markers of disease activity DAS28, CDAI, and tender joints count based on 28 joints ([TJC28] [Supplementary Figure S2]). The OPLS analysis was followed by multiple linear regression analyses between leptin and the above-mentioned markers after adjustment for sex, age, and BMI and no significant associations were found (Supplemental Table S1). Circulating resistin levels were unrelated to inflammation and disease activity markers in this cohort of patients with untreated newly diagnosed RA (Supplementary Figure S2).

## 4. Discussion

In this study, we show that plasma levels of both total and HMW adiponectin were associated with circulating levels of pro-inflammatory chemokines in a cohort of 70 patients with newly diagnosed untreated RA. Moreover, both circulating total and HMW adiponectin were associated with markers of inflammation in the same cohort. Circulating leptin and resistin levels were, on the other hand, unrelated to chemokines or markers of inflammation and disease activity in this cohort.

Adiponectin is an adipocytokine whose levels are elevated in both plasma and synovial fluid of patients with RA [6,7]. We have recently shown that adiponectin is increased in blood several years before the development of RA, and that recombinant adiponectin can stimulate PBMCs and FLS from non-inflamed individuals to produce several chemokines and cytokines involved in the pathogenesis of RA [17,18]. Taken together, these results raise the hypothesis that adiponectin is involved in the early phases of RA development by promoting inflammation both systemically and locally. Here, we tested the hypothesis that total and HMW adiponectin are associated with circulating chemokines in patients with RA. We also studied if circulating adiponectin was associated with markers of inflammation and RA disease activity. Therefore, we analyzed a cohort of 70 treatment-naive patients with newly diagnosed RA to avoid possible bias caused by anti-rheumatic treatment and long-term disease duration, which could affect the association between adiponectin and inflammation markers. In fact, DMARDs and glucocorticoids have an impact on both circulating adiponectin and chemokine levels, which precludes the use of treated patients in studies of association between levels of adiponectin and chemokines or clinical markers [27,34,35,36,37,38,39,40]. This might explain why a few previous studies failed to detect an association between adiponectin and inflammation or disease activity markers in RA [19,27].

We screened 15 chemokines in the plasma of 70 patients with untreated newly diagnosed RA and found that CXCL10, CXCL9, and CCL2 were positively associated with total adiponectin levels. CXCL10 and CXCL9 share the same receptor, CXCR3, which is predominantly expressed by activated T cells and regulates cell activation and trafficking [41]. Notably, in a subgroup of our cohort, it has been previously shown that plasma CXCL10 levels are positively associated with multiple disease activity markers of RA, such as DAS28-CRP, DAS28-ESR, CRP, and ESR [15]. The same study also showed that circulating CXCL9 was associated with ESR. Moreover, circulating CCL2 levels are elevated in patients with RA compared to controls and CCL2 increase has been reported as strongly related to the future incidence of RA in healthy individuals [12,42]. Additionally, CCR2, the receptor for CCL2, is a potent inducer of macrophage and monocyte recruitment to the site of inflammation [43]. Hence, our results show that adiponectin levels associated with chemokines that are closely linked to the pathophysiology and activity of RA.

In our cohort circulating levels of total adiponectin were associated with CRP and ESR after adjustment for sex, age, and BMI. Adiponectin levels were also associated with both DAS28-CRP and DAS28-ESR, but not with SJC, TJC, or CDAI. As DAS28 index includes CRP or ESR in its formula, there is reason to believe that the association between DAS28 and adiponectin was mainly driven by CRP and ESR. To the best of our knowledge, two previous studies have analyzed the association between circulating adiponectin and markers of inflammation and disease activity in patients with untreated newly diagnosed RA. A recent study has demonstrated that both total and HMW adiponectin associated with CRP, but not ESR or DAS28 in a cohort of 60 untreated newly diagnosed patients with RA [21]. In another study, no association was detected between circulating total adiponectin and markers of disease activity in 40 patients with untreated newly diagnosed RA [27]. The novelty of our work resides in the fact that we did a more comprehensive analysis of the inflammatory state of our cohort by measuring circulating chemokines as well as markers of inflammation and disease activity. Moreover, all our analyses were adjusted for potential confounders. In fact, BMI, age, and sex strongly correlate with adiponectin levels and can also affect markers of inflammation and disease activity, making it mandatory to adjust for them to have a clear picture of the association between adiponectin and markers of inflammation and disease activity [31,44,45,46].

Adiponectin circulates in blood in three different forms, whereof the HMW seems to be the form mediating the metabolic effects [20]. To determine whether HMW adiponectin was also the form exerting inflammatory functions involved in RA pathophysiology, we measured total and HMW adiponectin in our cohort. Both total and HMW adiponectin associated positively with CRP, ESR, and DAS28. This result might suggest that the HMW form drives the association between adiponectin and markers of inflammation in early RA. HMW adiponectin was also associated with CXCL9 but not with CXCL10 or CCL2, as was total adiponectin. Based on the effect size of the analyses, our cohort may be underpowered to detect a significant association between those chemokines and HMW adiponectin. Another possible explanation is that the association of total adiponectin with CXCL10 and CCL2 is mediated by another adiponectin form. However, one naturally arising limitation of the current study is that no analysis of low-molecular weight (LMW) or middle-molecular weight (MMW) adiponectin was performed due to lack of commercial products available to measure these forms. Without the assessment of all circulating forms of adiponectin, it is hard to draw a clear conclusion on the associations of the predominant form with chemokines, markers of inflammation, and disease activity.

Leptin and resistin are adipocytokines whose circulating levels are elevated in patients with RA and therefore they have been suggested as possible markers of disease activity [6,8]. However, the association between those adipocytokines and markers of inflammation and disease activity in patients with RA has been long debated [6,8,9,25]. In this study, we explored the relationship of leptin and resistin with 15 plasma chemokines as well as inflammation and disease activity markers in our early RA cohort. We found no associations between either of those adipocytokines with plasma chemokines or markers of inflammation and disease activity. It is noteworthy that most of the previous studies on the subject have been performed in patients with established RA, where both the treatment and the disease duration could have affected leptin and resistin levels [9,47]. Our analysis, free of confounders, showed that adiponectin was the only one of the measured adipocytokines to be associated with pro-inflammatory chemokines and disease activity markers.

This is an exploratory study with some limitations. First, the above-mentioned lack of measurement of plasma LMW and MMW adiponectin is a major limitation. Moreover, a larger cohort of patients with untreated RA should be analyzed to confirm the association between adipocytokines and chemokines or markers of inflammation and disease activity. In the future, clinical follow-up after treatment would provide information on how treatment affects adipocytokine levels and whether a higher baseline level of a certain adipocytokine associates with treatment outcomes. Another limitation of the study is that it does not have a control group of patients with other inflammatory joint diseases. A comparison between the association of adiponectin with chemokines and markers of inflammation in RA vs. other inflammatory joint diseases would have provided information on the specificity of our findings.

## 5. Conclusions

By studying a clinically well-characterized cohort of patients with untreated newly diagnosed RA we have shown an association between circulating adiponectin and pro-inflammatory chemokines involved in the pathogenesis of RA as well as markers of inflammation, whereas leptin and resistin showed no such associations.

## Figures and Tables

**Table 1:** Demographic and clinical characteristics of the cohort of patients with untreated newly diagnosed rheumatoid arthritis (RA).

Characteristic	Early RA (no = 70)
Women, no (%)	47 (69)
Age, yr	55 (42–64)
BMI, kg/m^2^	25 (23–28)
CRP, mg/L	9 (4–31)
ESR, mm/hour	24 (12–38)
SJC28	9 (5–12)
TJC28	9 (4–13)
DAS28-CRP	5 (4–6)
DAS28-ESR	5 (5–6)
CDAI	28 (22–38)
ACPA+, no (%)	57 (81%)
RF+, no (%)	48 (69%)
Symptom Duration (months)	5 (3–8)
Smoking, no (%) *	15 (22)

Data shown as median (interquartile range), unless noted as no (%) in which case they are shown as number (percentage). * Defined as current daily smoking. Abbreviations: BMI, body-mass index; CRP, C-reactive protein; ESR, erythrocyte sedimentation rate; SJC28, swollen joint counts of 28; TJC28 tender joint counts of 28; DAS28, disease activity score in 28 joints; CDAI, Clinical Disease Activity Index; ACPA, anti–citrullinated protein antibody; RF, rheumatoid factor.

**Table 2:** Levels of plasma adipocytokines and chemokines.

Protein	Early RA/(no = 70)
Total Adiponectin (mg/L)	6.0 (4.0–10.3)
HMW Adiponectin (mg/L)	3.0 (1.5–5.2)
Leptin (ng/mL)	13.2 (4.7–35.3)
Resistin (ng/mL) *	10.1 (7.8–13.4)
CXCL8/IL-8	489 (162–1001)
CXCL10/IP-10	1024 (748–1859)
CCL17/TARC	301 (215–516)
CCL2/MCP-1	960 (746–1195)
CCL5/RANTES	17,538 (13,354–22,134)
CCL3/MIP-1α *	29 (14–56)
CXCL9/MIG *	245 (89–813)
CXCL5/ENA-78	547 (311–762)
CCL20/MIP-3α *	25 (2–74)
CXCL1/GROα *	214 (147–341)
CXCL11/I-TAC	985 (736–1446)
CCL4/MIP-1β *	44 (28–72)
CCL22/MDC	466 (356–620)
CXCL13/BCL	490 (205–990)
CCL11/Eotaxin	732 (628–1056)

Data shown as median (interquartile range). All units are in pg/mL, unless otherwise noted. * Resistin was under detection limit in 1% of samples, CCL3/MIP-1α in 15%, CXCL9/MIG in 14%, CCL20/MIP-3a in 35%, CXCL1/GROα in 17%, and CCL4/MIP-1β in 11%.

## Data Availability

All relevant data are within the manuscript and its supporting information files.

## Supplementary Materials

The following are available online at https://www.mdpi.com/2218-273X/11/2/325/s1, Figure S1: OPLS loadings column plot of the association of leptin and resistin with plasma chemokines, Figure S2: OPLS loadings column plot of the association of leptin and resistin with clinical markers of disease activity, Table S1: Linear regression analysis of leptin with plasma chemokines and clinical parameters, Table S2: Linear regression analysis of resistin with plasma chemokines.
