# Supplementary material for: Adipocytokines in Untreated Newly Diagnosed Rheumatoid Arthritis: Association with Circulating Chemokines and Markers of Inflammation
Source: Biomolecules. 2021 Feb 21;11(2):325. doi: 10.3390/biom11020325 (PMC7924659; doi:10.3390/biom11020325)
Supplement: Supplementary file 1 [file biomolecules-11-00325-s001.zip › Suplementary Tables Vasileiadis et al.pdf]

Table S1. Linear regression analysis of leptin with plasma chemokines and clinical parameters.

Characteristic	Leptin	
	**p-value**	**β coef**
CXCL9/MIG*	0.512	0.130
CCL3/MIP-1α*	0.351	0.186
CXCL1/GROα*	0.192	0.256
CXCL11/I-TAC	0.838	0.038
DAS28-ESR	0.588	0.098
CDAI	0.406	0.151
DAS28-CRP	0.414	0.149
TJC28	0.341	0.175

Multiple linear regression adjusted for sex, age and BMI. Only variables associated with plasma leptin in the OPLS analysis were tested for significance with linear regression analyses.

Abbreviations: DAS28, disease activity score in 28 joints; *ESR,* erythrocyte sedimentation rate; *CDAI,* Clinical Disease Activity Index; *CRP,* C-reactive protein; *TJC28* tender joint counts of 28.

* CCL3/MIP-1α was undetectable in 15% of samples, CXCL9/MIG in 14%, CXCL1/GROα in 17% and CCL4/MIP-1β in 11%.

Table S2. Linear regression analysis of resistin with plasma chemokines

Characteristic	Resistin	
	**p-value**	**β coef**
CXCL1/GROα*	0.859	0.025
CCL3/MIP-1α*	0.576	0.078
CCL4/MIP-1β*	0.462	0.103
CXCL8/IL-8	0.157	0.196

Multiple linear regression adjusted for sex, age and BMI. Only variables associated with plasma resistin in the OPLS analysis were tested for significance with linear regression analyses.

* CXCL1/GROα was undetectable in 17% of samples, CCL3/MIP-1α in 15% and CCL4/MIP-1β in 11%.
