# Supplementary material for: Adipocytokines in Untreated Newly Diagnosed Rheumatoid Arthritis: Association with Circulating Chemokines and Markers of Inflammation
Source: Biomolecules. 2021 Feb 21;11(2):325. doi: 10.3390/biom11020325 (PMC7924659; doi:10.3390/biom11020325)
Supplement: Supplementary file 1 [file biomolecules-11-00325-s001.zip › supplementary figures.pdf]

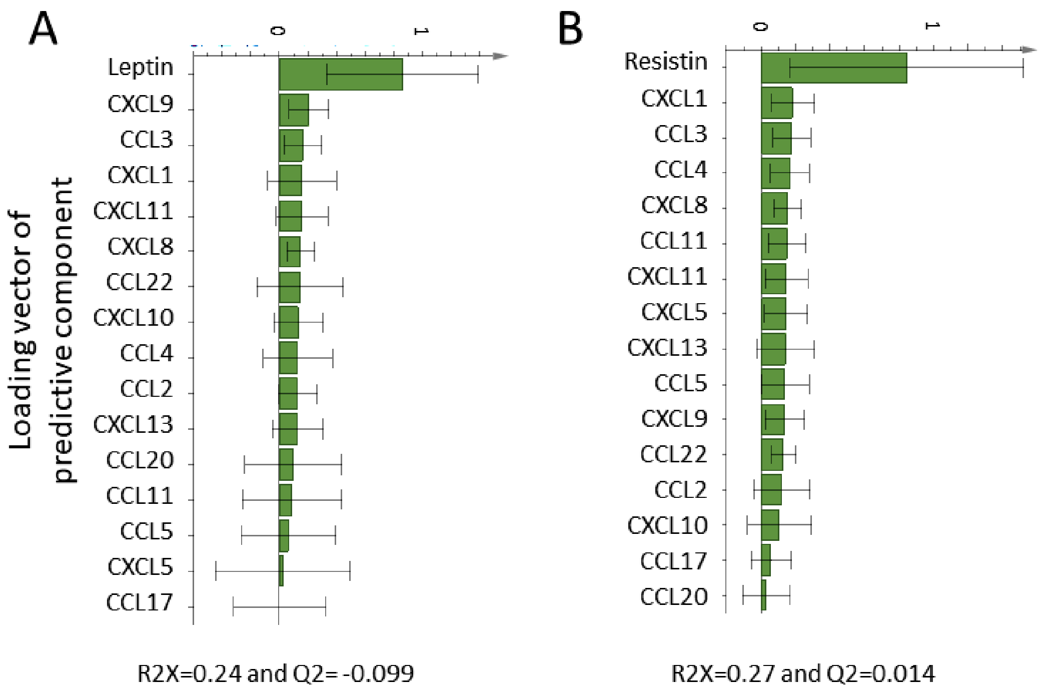

Supplementary Figure 1. OPLS loadings column plot of the association of leptin and resistin with plasma chemokines. OPLS models depicting the association of leptin (A) and resistin (B) with plasma chemokines. X-variables represented by a bar pointing in the same direction as the y-variable (adipocytokine) were associated with higher leptin or resistin levels, whereas x-variables represented by a bar pointing in the opposite direction were related to lower leptin or resistin. Only variables associated with plasma leptin/resistin in the OPLS analysis were tested for significance with linear regression analyses. *Abbreviation*: OPLS, orthogonal projection to latent structures.

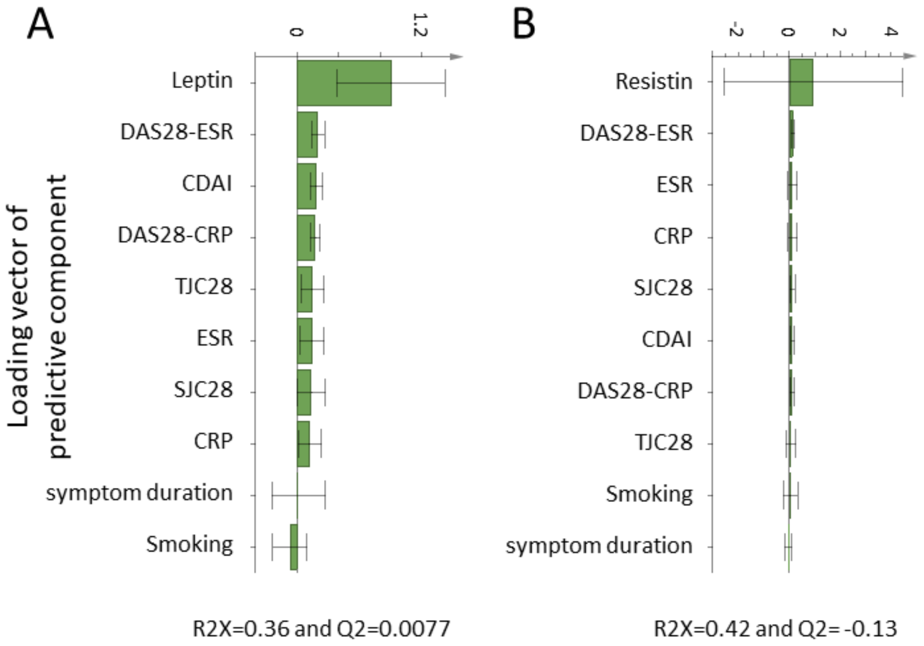

Supplementary Figure 2. OPLS loadings column plot of the association of leptin and resistin with clinical markers of disease activity. OPLS models depicting the association of leptin (A) and resistin (B) with clinical markers of disease activity. X-variables represented by a bar pointing in the same direction as the y-variable (adipocytokine) were associated with higher leptin or resistin levels, whereas x-variables represented by a bar pointing in the opposite direction were related to lower leptin or resistin. Only variables associated with plasma leptin/resistin in the OPLS analysis were tested for significance with linear regression analyses. *Abbreviations*: CDAI, clinical disease activity index; CRP, C-reactive protein; DAS28, disease activity score in 28 joints; ESR, erythrocyte sedimentation rate; OPLS, orthogonal projection to latent structures; SJC28, swollen joint counts of 28; TJC28, tender joint counts of 28.
